# Mycorrhiza stimulates root-hair growth and IAA synthesis and transport in trifoliate orange under drought stress

**DOI:** 10.1038/s41598-018-20456-4

**Published:** 2018-01-31

**Authors:** Chun-Yan Liu, Fei Zhang, De-Jian Zhang, AK Srivastava, Qiang-Sheng Wu, Ying-Ning Zou

**Affiliations:** 1 College of Horticulture and Gardening, Yangtze University, Jingzhou, Hubei 434025 China; 2 Institute of Root Biology, Yangtze University, Jingzhou, Hubei 434025 China; 3 ICAR-Central Citrus Research Institute, Amravati Road, Nagpur, 440033 Maharashtra India; 4 Department of Chemistry, Faculty of Science, University of Hradec Kralove, Hradec Kralove, 50003 Czech Republic

## Abstract

Root-hair growth and development regulated by soil microbes is associated with auxin. In this background, we hypothesized that mycorrhizal fungal inoculation induces greater root-hair growth through stimulated auxin synthesis and transport under water stress conditions. Trifoliate orange (*Poncirus trifoliata*) was inoculated with an arbuscular mycorrhizal (AM) fungus (*Funneliformis mosseae*) under well-watered (WW) and drought stress (DS) for 9 weeks. Compared with non-AM seedlings, AM seedlings displayed significantly higher density, length, and diameter of root hairs and root indoleacetic acid (IAA) level, whereas lower total root IAA efflux, regardless of soil moisture status. Root *PtYUC3* and *PtYUC8* involved in IAA biosynthesis were up-regulated by mycorrhization under WW and DS, whereas AM-modulated expression in *PtTAA1*, *PtTAR2*, *PtYUC4*, and *PtYUC6* depended on status of soil moisture. Mycorrhizal inoculation down-regulated the transcript level of root auxin efflux carriers like *PtPIN1* and *PtPIN3*, whereas significantly up-regulated the expression of root auxin-species influx carriers like *PtABCB19* and *PtLAX2* under DS. These results indicated that AMF-stimulated greater root-hair growth of trifoliate orange under DS that is independent on AMF species is related with mycorrhiza-modulated auxin synthesis and transport, which benefits the host plant to enhance drought tolerance.

## Introduction

Drought stress (DS), one of the most frequently occurring abiotic stresses, often threatens the sustainability of agriculture through wide range of impacts on crop growth and production^1^. Arbuscular mycorrhizal (AM) fungi (AMF), one of soil inhabiting fungi, can form the symbiotic association with roots of higher plants, called AM symbiosis. The symbiosis is characterized by increased water and nutrient uptake diverted from the soil to the fungal partner, and in return receives the photosynthate supply from the host plant to the AMF^2^. Studies in the past showed that AMF strongly enhanced drought tolerance of host plants via varied mechanisms viz., direct absorption of water by mycorrhizal extraradical hyphae at the rate of 375–760 nL water/h, contributing upto 20% of total water absorbed by plant roots^3^,  improvement in osmotic adjustments^4,5^, enhancement in antioxidant profile coupled with enhanced efflux of hydrogen peroxide into rhizosphere^6–8^, and facilitating the formation of soil water-stable aggregates by both glomalin and mycorrhizal  hyphae^9^.

Root hairs that are tubular outgrowths from root-specific epidermis, function as water channels in plants^10^. It estimates that root hairs comprise as much as two third of the total root surface area^11^. Inoculation with AMF generally showed an increase in root hair density of the host plants^12^. Recently, Zou *et al*.^13^ reported that under DS conditions, an AM fungus, *Diversispora versiformis*, significantly increased the root hair density and length in trifoliate orange, without any influence on root hair diameter. Such responses on root hair density and lengths under mycorrhization potentially provide an increased surface area to facilitate mycorrhized plants absorb more water and nutrients^11^. Li *et al*.^14^ used a bald root barley and its wild type to evaluate the relative importance of AMs under irrigated versus DS conditions. Their results showed that AMs and root hairs collectively improved P-uptake in promoting the plant growth, plant water relations or photosynthetic capacity under DS, thereby, providing an evidence for enhancing drought tolerance of host plant under DS. The magnitude of plant responses to mycorrhization is considered highly dependent on the compatibility between AMF species and host plant species^15^. It is still not clear whether mycorrhizas could play a role in root hair growth, with an exception of *D*. *versiformis*^14^.

The mechanisms regarding mycorrhizal effects on root-hair growth of host plants are unknown. It is well documented that root-hair initiation is manipulated by two different pathways, viz., developmental pathway and the environmental/hormonal pathway^16^. In roots, a variety of of phytohormones, like auxin, ethylene, jasmonic acid, brassinosteroid, and strigolactone participate in root-hair growth and development, but auxin is most extensively studied^17–19^. Auxin is primarily synthesized at the shoot apex, transported to the root tip by vascular tissues of the stem, finally moves in a basipetal orientation towards the elongation zone through root peripheral tissues^20^. Such auxin efflux at the root apex is mainly controlled by various Pin-formed (PIN) auxin efflux carriers, besides AUXIN RESISTANT 1/LIKE AUX1 (AUX1/LAX) auxin influx carriers and some members of the ATP-BINGING CASSETTE B (ABCB) transporters (auxin efflux proteins)^21,22^. Besides auxin transport, auxin synthesis is controlled by a number of genes, such as tryptophan aminotransferase (*TAA*), tryptophan aminotransferase related (*TAR*), flavin monooxygenase-like enzyme (*YUC*), etc.^23^.

Trifoliate orange (*Poncirus trifoliata* L. Raf.) is a widely used rootstock in citriculture in Southeast Asia, the root configuration of which is characterized by distinctively few and short root hairs which, accompanied with highly drought-sensitive nature^24^. Based on our previous results^13^, we hypothesized that mycorrhizal inoculation with *Funneliformis mosseae* could induce greater root-hair growth of trifoliate orange through enhanced auxin synthesis and transport under DS for enhanced drought tolerance. To confirm this hypothesis, trifoliate orange seedlings were inoculated with *Funneliformis mosseae* and subsequently exposed to well-watered (WW) and DS conditions. The responses were evaluated through root-hair morphology, root auxin concentration, root auxin effluxes, and relative expression of root auxin relevant genes.

## Results

### Mycorrhizal colonization of roots

No mycorrhizal colonization was observed in non-AM roots. AMF-inoculated seedlings showed 55.6–61.4% of root mycorrhizal colonization (Table 1). As much as 9.4% reduction in root mycorrhizal colonization was observed under DS than under WW.

**Table 1:** Effects of an arbuscular mycorrhizal fungus (AMF), *Funneliformis mosseae*, on plant growth performance of trifoliate orange (*Poncirus trifoliata*) seedlings exposed to well-watered (WW) and drought stress (DS).

Treatments	Root mycorrhizal colonization (%)	Plant height (cm)	Stem diameter (mm)	Leaf number (#/plant)	Biomass (g FW/plant)	
					Shoot	Root
WW − AMF	0c	25.1 ± 1.2c	4.28 ± 0.15b	23 ± 2c	1.93 ± 0.06c	1.96 ± 0.13c
WW + AMF	61.4 ± 1.1a	45.9 ± 0.6a	5.23 ± 0.29a	32 ± 1a	4.55 ± 0.14a	3.06 ± 0.21a
DS − AMF	0c	17.9 ± 1.6d	3.45 ± 0.19c	19 ± 1d	1.30 ± 0.03d	1.67 ± 0.09d
DS + AMF	55.6 ± 1.2b	39.5 ± 2.5b	4.40 ± 0.22b	29 ± 2b	3.64 ± 0.12b	2.61 ± 0.09b

Data (means ± SD, *n* = 4) followed by different letters in the column indicate significant differences (*P* < 0.05) between treatments.

### Plant growth

Plant growth traits, including plant height, stem diameter, leaf number, and shoot and root biomass were adversely affected by DS treatment, as compared with WW treatment, regardless of AM or non-AM seedlings (Table 1). On the other hand, AM seedlings showed significantly higher these plant growth-related traits than non-AM seedlings, irrespective of WW or DS condition.

### Root-hair features

**Figure 1:**
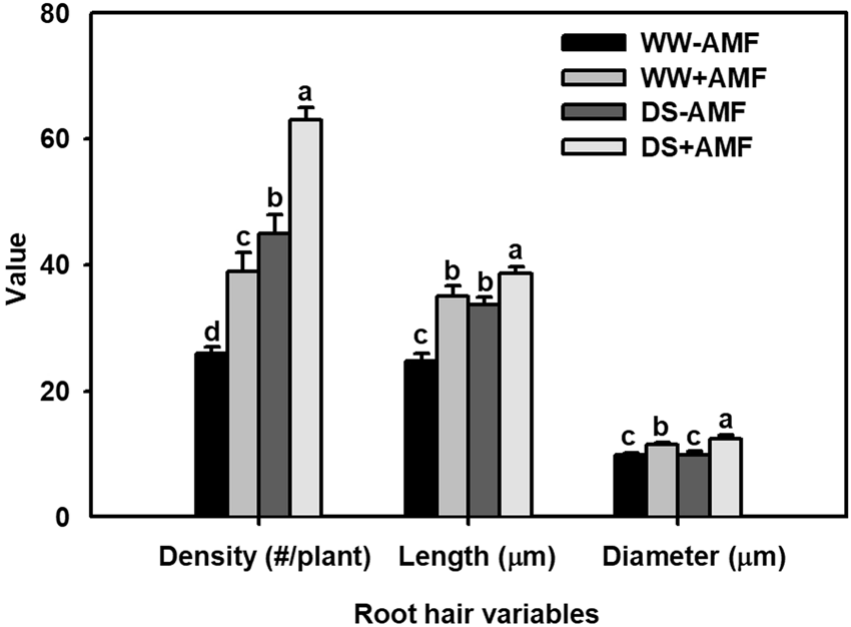
Effects of an arbuscular mycorrhizal fungus (AMF), *Funneliformis mosseae*, on average density, length, and diameter of root hairs of trifoliate orange (*Poncirus trifoliata*) seedlings exposed to well-watered (WW) and drought stress (DS). Data (means ± SD, *n* = 4) followed by different letters above the bars indicate significant differences (*P* < 0.05) between treatments.

Length, diameter and density of root hairs were significantly increased by DS treatment, in comparison with WW treatment (Fig. 1). AM seedlings displayed better root hair features than non-AM seedlings under both WW and DS, in a range of 41% and 15% higher for root hair length, 50% and 40% higher for root hair density, and 16% and 25% higher for root hair diameter, respectively.

### Root IAA concentration

**Figure 2:**
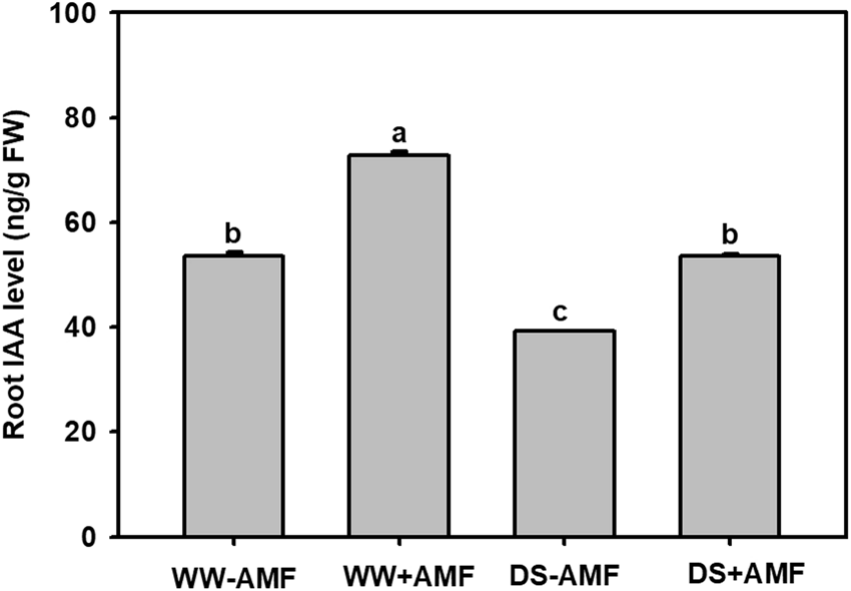
Effects of an arbuscular mycorrhizal fungus (AMF), *Funneliformis mosseae*, on root IAA concentration of trifoliate orange (*Poncirus trifoliata*) seedlings exposed to well-watered (WW) and drought stress (DS). Data (means ± SD, *n* = 4) followed by different letters above the bars indicate significant differences (*P* < 0.05) between treatments.

Concentration of root indole-3-acetic acid (IAA) was significantly reduced by DS treatment, as compared to WW treatment (Fig. 2). AM seedlings exhibited significantly higher root IAA concentration than non-AM seedlings by 36% and 37% under WW and DS condition, respectively.

### Total root IAA efflux

**Figure 3:**
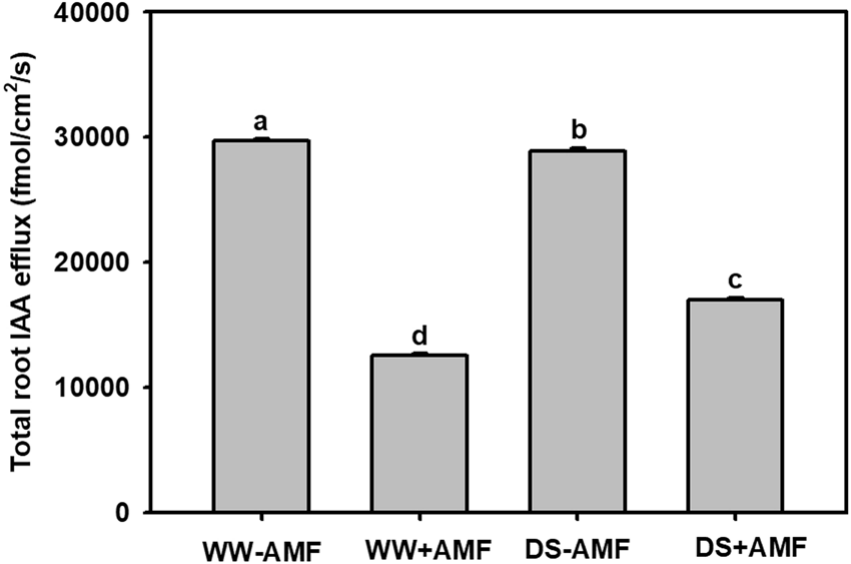
Effects of an arbuscular mycorrhizal fungus (AMF), *Funneliformis mosseae*, on root IAA effluxes of trifoliate orange (*Poncirus trifoliata*) seedlings exposed to well-watered (WW) and drought stress (DS). Data (means ± SD, *n* = 4) followed by different letters above the bars indicate significant differences (*P* < 0.05) between treatments.

An IAA efflux was observed in trifoliate orange from the root to the rhizosphere, regardless of WW or DS treatment. The DS treatment produced a significant reduction in total root IAA efflux by 3% in non-AM seedlings and an increase by 35% in AM seedlings (Fig. 3). AMF inoculation conferred a significant reduction in total root IAA efflux by 58% and 41% under WW and DS, respectively, in relative to non-AMF treatment.

### Root IAAO activity

**Figure 4:**
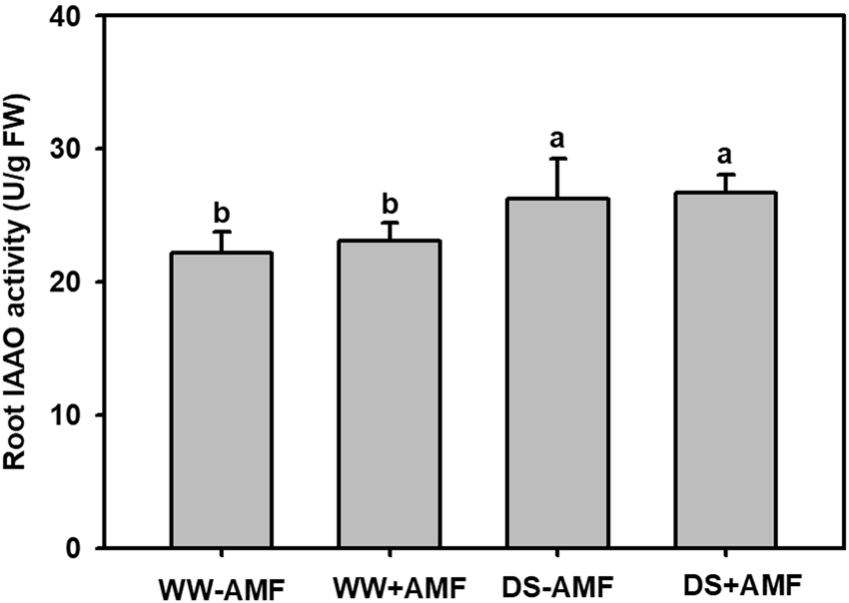
Effects of an arbuscular mycorrhizal fungus (AMF), *Funneliformis mosseae*, on root IAAO activity of trifoliate orange (*Poncirus trifoliata*) seedlings exposed to well-watered (WW) and drought stress (DS). Data (means ± SD, *n* = 4) followed by different letters above the bars indicate significant differences (*P* < 0.05) between treatments.

Compared with WW treatment, root IAA oxidase (IAAO) activity was significantly increased by DS treatment in AM or non-AM seedlings (Fig. 4). There were no significant difference in root IAAO activity between AM and non-AM seedlings exposed to both WW and DS.

### Transcript levels of IAA relevant genes in roots

**Figure 5:**
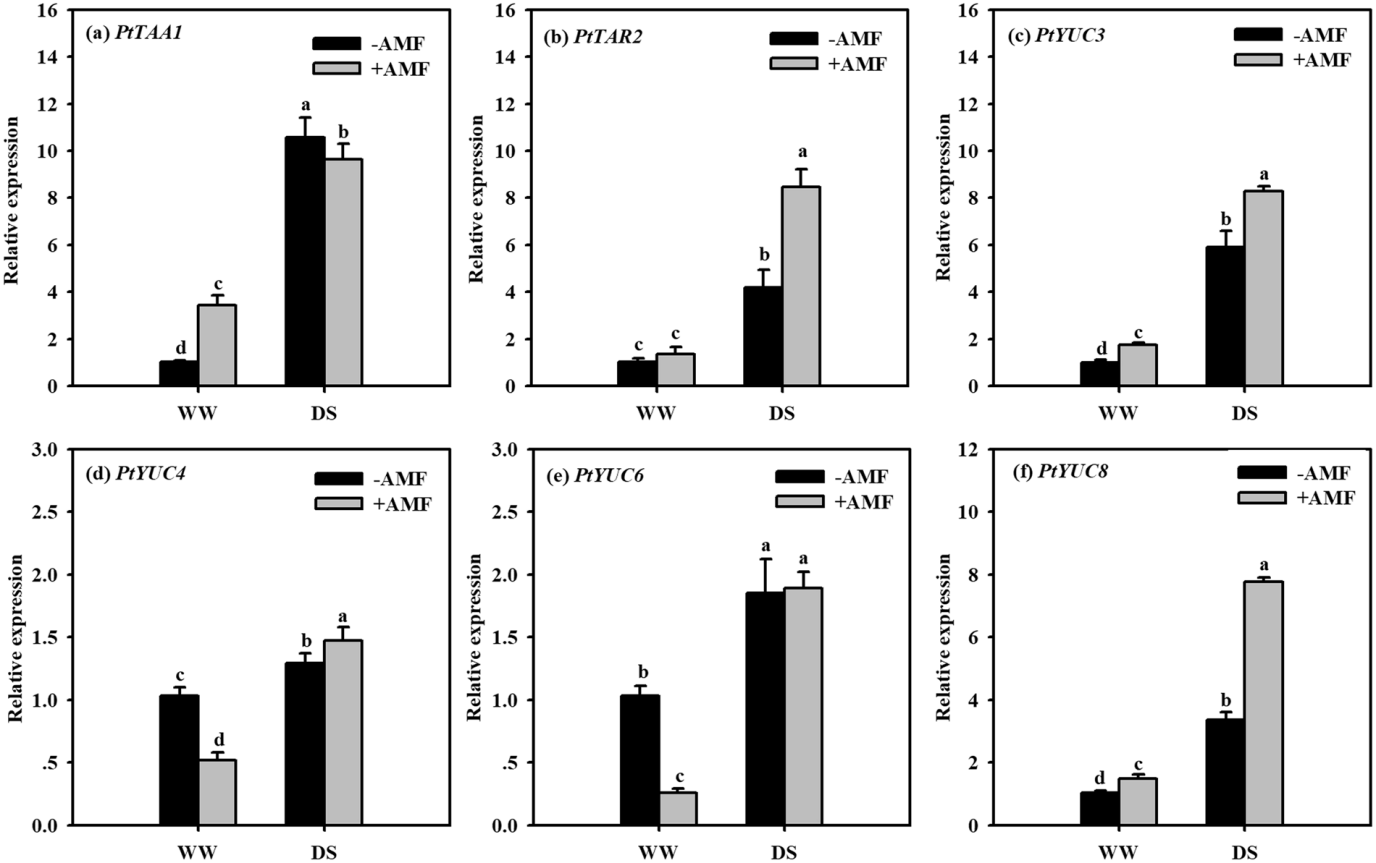
Effects of an arbuscular mycorrhizal fungus (AMF), *Funneliformis mosseae*, on relative expression of root IAA synthetic genes, *PtTAA1* (**a**), *PtTAR2* (**b**), *PtYUC3* (**c**), *PtYUC4* (**d**), *PtYUC6* (**e**), and *PtYUC8* (**f**) in trifoliate orange (*Poncirus trifoliata*) seedlings exposed to well-watered (WW) and drought stress (DS). Data (means ± SD, *n* = 4) followed by different letters above the bars indicate significant differences (*P* < 0.05) between treatments.

A considerable enhancement in transcript levels of IAA relevant genes was observed in roots under DS treatment, compared with WW treatment (Fig. 5a–f). Under WW condition, AMF inoculation showed a significant increase in transcript level of *PtTAA1*, *PtYUC3*, and *PtYUC8* by 239%, 74%, and 45%, respectively, compared with non-AMF inoculation. On the other hand, AM seedlings displayed 102%, 41%, 14%, and 131%, respectively, higher transcript levels of *PtTAR2*, *PtYUC3*, *PtYUC4* and *PtYUC8* and 9% lower expression of *PtTAA1*, relative to non-AM seedlings under DS.

### Transcript levels of IAA carrier genes in roots

**Figure 6:**
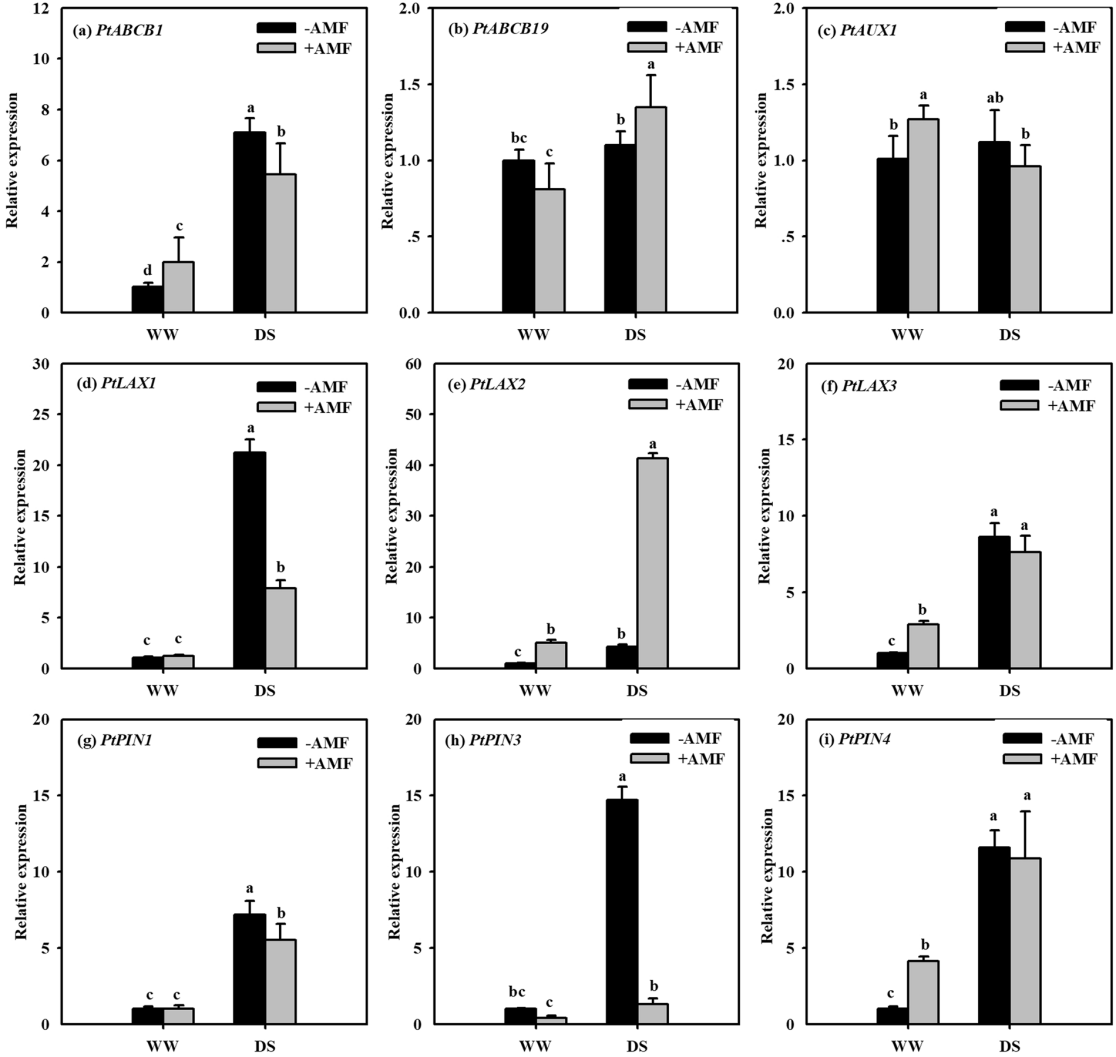
Effects of an arbuscular mycorrhizal fungus (AMF), *Funneliformis mosseae*, on relative expression of root IAA-species carriers, *PtABCB1* (**a**), *PtABCB19* (**b**), *PtAUX1* (**c**), *PtLAX1* (**d**), *PtLAX2* (**e**), *PtLAX3* (**f**), *PtPIN1* (**g**), *PtPIN3* (**h**), and *PtPIN4* (**i**) in trifoliate orange (*Poncirus trifoliata*) seedlings exposed to well-watered (WW) and drought stress (DS). Data (means ± SD, *n* = 4) followed by different letters above the bars indicate significant differences (*P* < 0.05) between treatments.

Under WW condition, AMF inoculation produced no change in the relative expression level of *PtABCB1*, *PtABCB19* and *PtLAX1*, but significantly up-regulated the expression of *PtAUX1*, *PtLAX2* and *PtLAX3* by 26%, 394%, and 191%, respectively (Fig. 6a–i). Under DS condition, the transcript level of genes such as *PtAUX1*, *PtLAX3*, and *PtPIN4* remained unaffected by AMF inoculation, whereas the significant up-regulation at the transcript level of *PtABCB19* and *PtLAX2* genes (23% and 880%, respectively) and down-regulation in *PtABCB1*, *PtLAX1*, *PtPIN1* and *PtPIN3* genes (23%, 63%, 23% and 91%, respectively) were observed under AMF inoculation versus under non-AMF treatment.

### Transcript levels of root EXPAs

**Figure 7:**
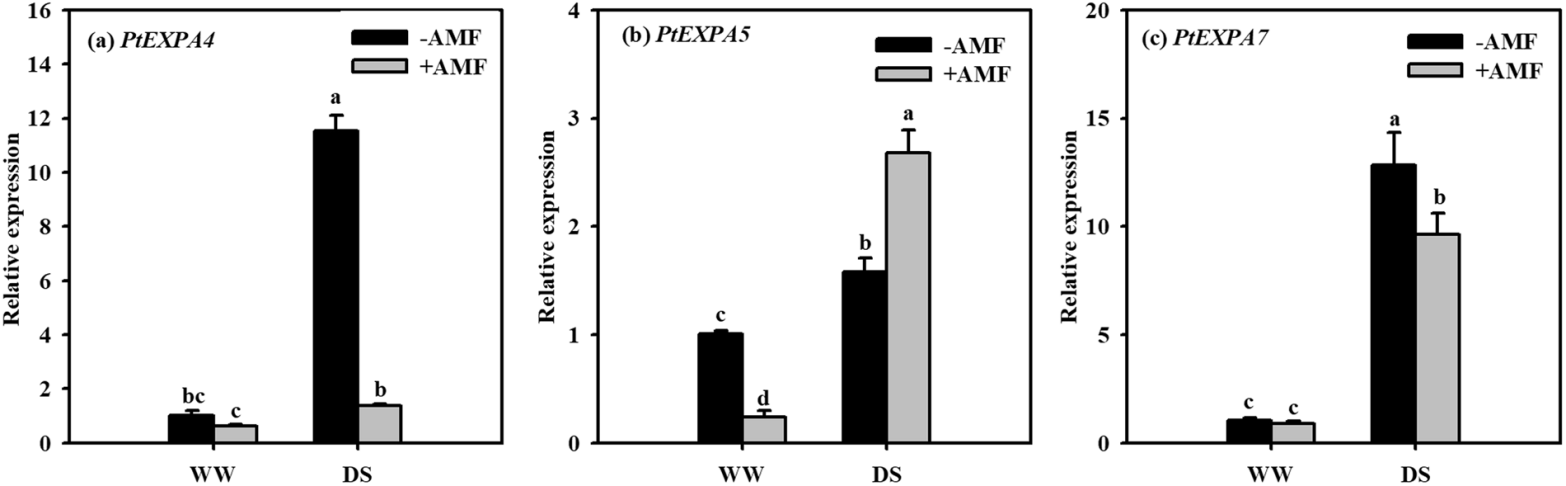
Effects of an arbuscular mycorrhizal fungus (AMF), *Funneliformis mosseae*, on relative expression of root *PtEXPA4* (**a**), *PtEXPA4* (**b**), and *PtEXPA7* (**c**) in trifoliate orange (*Poncirus trifoliata*) seedlings exposed to well-watered (WW) and drought stress (DS). Data (means ± SD, *n* = 4) followed by different letters above the bars indicate significant differences (*P* < 0.05) between treatments.

Root *EXPAs* such as *PtEXPA4*, *PtEXPA5*, and *PtEXPA7* genes were significantly up-regulated by DS, compared with WW, irrespective of AM or non-AM-seedlings (Fig. 7a–c). Three *PtEXPA* genes expressed differential responses to AMF inoculation. Under WW, AMF inoculation down-regulated the expression of *PtEXPA5* only by 76% (Fig. 7b). Nevertheless, under DS, AMF treatment up-regulated root *PtEXPA5* gene expression by 70% and down-regulated *PtEXPA4* and *PtEXPA7* gene expression by 88% and 25%, respectively (Fig. 7a,c).

## Discussion

In this study, inoculation with *F*. *mosseae* showed a significant increase in length, density, and diameter of root hairs in trifoliate orange under DS. These observations are in agreement with the study carried out by Zou *et al*.^13^ in trifoliate orange colonized by *Diversispora versiformis* under DS. It also suggested that mycorrhiza-stimulated root hair growth in trifoliate orange is independent of AMF species. Better root hair growth in mycorrhizal trifoliate orange plants under DS provides a much higher nutrient foraging ability for the inoculated plants to absorb more water and nutrients from mycorrhizosphere, eventually alleviating negative effects of drought on plants^25^.

The present study showed that mycorrhizal inoculation produced a remarkably increased root IAA level in trifoliate orange seedlings exposed to either WW or DS, in accordance with our previous study in trifoliate orange seedlings colonized by *Claroideoglomus etunicatum*, *D*. *versiformis*, *F*. *mosseae*, and *Rhizoglomus intraradices*^12^. Auxin is now considered as a key regulator in the whole process of root-hair initiation, growth and development^18,19,26^. Any increase in the root IAA accumulation under mycorrhization, thereby, would benefit root-hair growth and plant growth performance.

Auxin is transported from one cell to another cell, following a strict directionality in uptake and efflux of carrier proteins involved^23^. Auxin efflux is regulated through members of PIN family, the sequence of which encodes a family of auxin efflux carriers^22^. Our study revealed that mycorrhizal seedlings displayed lower root IAA efflux than non-mycorrhizal seedlings under either WW or DS treatment. Under DS, mycorrhizal treatment down-regulated the transcript level of root *PtPIN1* and *PtPIN3*, but not *PtPIN4*, indicating a potential reduction in the amount of auxin efflux and finally resulting in an elevated accumulation of root IAA in AM plants over non-AM plants. Tromas and Parrot-Rechenmann^22^ reported that *PIN1*, *PIN3*, and *PIN4* were located in stele cells, collectively responsible for auxin flow towards the quiescent center (QC), close to root tip for auxin reflux. Therefore, the lower expression level of *PtPIN1* and *PtPIN3* in AM versus non-AM plants exposed to DS is speculated to decrease the auxin flow towards the QC, thereby, reducing the auxin reflux^27^ and inducing greater auxin accumulation in the root-hair zones to stimulate root-hair growth. In fact, auxin reflux is associated with the five PIN proteins (PIN1, PIN2, PIN3, PIN4 and PIN7)^27,28^. Further studies are needed to decode the functioning of the PIN family on AMF-induced root hair modification, especially under DS condition.

It is well known that YUC (YUCCA encoding a flavin monooxygenase) and TAA/TAR (Tryptophan Aminotransferase of Arabidopsis) are two families of genes associated with auxin biosynthesis in plants^29^. In our work, the expression of root *PtTAA1*, *PtYUC3*, and *PtYUC8* under WW and root *PtTAR2*, *PtYUC3*, *PtYUC4*, and *PtYUC8* under DS was up-regulated by AMF inoculation, relative to non-AMF treatment. In the indole-3-pyruvic acid (IpyA) pathway, TAA1 and its close homologue, *PtTAR2* convert L-Trp into IpyA, and YUC enzymes synthesize IAA from IpyA^30^. Our present study showed a different capacity in conversation of L-Trp into IpyA by mycorrhization from WW to DS. And, *PtYUC3* and *PtYUC8* were jointly activated by mycorrhization, regardless of WW or DS, implying the high responsiveness of the two genes to mycorrhization.

Initiation and growth of root hairs require loosening of cell-wall components, mediated by cell wall-loosening expansin proteins (EXPs), represented by two major EXP subgroups, viz., EXPA and EXPB^31^. In our study, the transcript level of root *PtEXPA4* and *PtEXPA7* genes was not affected by AMF inoculation under WW, but the relative expression of root *PtEXPA5* was down-regulated. Under DS, AM seedlings were characterized by relatively higher transcript level of root *PtEXPA5* and lower transcript level of root *PtEXPA4* and *PtEXPA7* genes, indicating that AMF-mediated expression of root *PtEXPAs* is strongly dependent on soil moisture status. Inactivation or down-regulation in the expression of root *PtEXPAs* upon mycorrhization (except an up-regulated expression of root *PtEXPA5* under DS) further suggested that the member of root EXPAs is not stimulated by AMF to initiate growth elongation of root hairs.

Cell-to-cell auxin transport is dependent on two families of auxin-species carrier proteins, viz., ABCB family and AUX1/LAX family of influx carriers^32^, besides PIN carriers. ABCB auxin transporters are involved in the polar transport of IAA in plants, whilst ABCB1 and ABCB19 operate long-distance IAA-transport^33^. The AUX1/LAX family of PM permeases possess H^+^-symport activity to transport auxin into the cells^34^. Our work showed no changes in the expression of root *PtABCB1* and *PtABCB19* genes in response to mycorrihization under WW. Nevertheless, the root *PtABCB1* transcript level was decreased and transcript level of root *PtABCB19* was increased under DS in response to AMF inoculation. These observations warranted that mycorrhizal inoculation only induced the up-expression of root *PtABCB19* under DS to accelerate long-distance auxin-transport. A considerably higher transcript level of root *PtAUX1*, *PtLAX2*, and *PtLAX3* genes under WW was observed in AM than in non-AM seedlings. And, a higher expression level of root *PtLAX2* and lower expression level of root *PtLAX1* were observed in AM seedlings than in non-AM seedlings under DS, suggesting that these auxin carrier proteins, especially PtLAX2 responded well to mycorrhization for cell-to-cell auxin transport under DS.

IAAO is usually involved in auxin catabolism and negatively correlated with IAA levels, thereby, regulating the concentration of IAA^35^. In our work, DS treatment induced a higher root IAAO activity in both AM and non-AM seedlings, thereby, leading to the lower root IAA level in DS-treated seedlings. AMF-inoculation did not alter the root IAAO activity under both WW and DS, implying no relation between AMF-induced IAA increase and IAA catabolism.

In short, the present study confirmed our proposed hypothesis that mycorrhizal inoculation induced greater root-hair growth of trifoliate orange, closely associated with auxin pathway under DS, where mycorrhiza activated the auxin relevant genes (*PtYUC3* and *PtYUC8*), up-regulated the auxin-species influx carrier genes (*PtABCB19* and PtLAX2), and down-regulated the auxin-species efflux carrier genes (*PtPIN1* and *PtPIN3*).

## Methods

### Plant culture

Four-leaf-old trifoliate orange seedlings grown in autoclaved sands without mycorrhization were transplanted into a 4.5-L capacity plastic pot each supplied with 4.0 kg of autoclaved (0.11 Mpa, 121 °C, 2 h) soil and sand mixture (1:1, v/v). Subsequently, 200 g inoculums (~4000 spores) of *Funneliformis mosseae* (Nicol. & Gerd.) Schüßler & Walker (BGC XZ02A) were inoculated into each pot. The AM fungus was made available by the Bank of Glomeromycota in China and propagated with white clover and spores identified in pot culture only. Mycorrhizal inoculum contained spores (20 spores/g), sands, fungal mycelium, and root fragments. In case of the non-AM fungal treatment, the same amount of autoclaved mycorrhizal inoculum was applied, along with a 2-mL filtrate (25 μm) of the inoculum to maintain similar microflora population, except the AM fungus. The experiment was conducted during April 6 – August 24, 2015 under glasshouse conditions (photosynthetic photon flux density is 880 μmol/m^2^/s, day/night temperature 28/21 °C, and relative humidity 85%) in the campus of Yangtze University, Hubei, China.

AM and non-AM seedlings were kept at 75% of maximum water holding capacity of the soil (soil WW status) for 11 weeks. Afterwards, half of the seedlings were still maintained under WW status for 9 weeks, and the other half seedlings were exposed to 55% of maximum water holding capacity (soil DS status) of the soil for 9 weeks. Soil water levels in the pots were measured daily by weighing, and the amount of water lost was supplied to maintain the designated soil water levels.

### Experimental design

The experiment consisted of four treatments with a completely randomized block arrangement: i. the seedlings inoculated with *F*. *mosseae* under WW (WW + AMF), ii. the seedlings inoculated without *F*. *mosseae* under WW (WW-AMF), iii. the seedlings inoculated with *F*. *mosseae* under DS (DS + AMF), and iv. the seedlings inoculated without *F*. *mosseae* under DS (DS-AMF). Each treatment had four replicates, for a total of 16 pots, each pot having 3 seedlings.

### Variable determination

#### Root mycorrhizal colonization

As many fifteen 1-cm-long root segments per seedlings were cleared by 10% KOH solution at 95 °C for 1.5 h and then stained with 0.05% trypan blue in lactophenol for 5 min^36^. Root mycorrhizal colonization was calculated as the percentage of mycorrhizal infected root lengths against total observed root lengths.

#### Plant growth

Plant growth-related parameters like plant height, stem diameter, and leaf number per plant were determined in all the seedlings. After harvested, the seedlings were divided into shoots and roots, to measure their fresh weight.

#### Root hairs

Eight 1.5-cm-long root hair zones at 3 cm away from the root tip in tap root and 1^st^-, 2^nd^-, and 3^rd^-order lateral roots were selected, fixed by 2.5% glutaraldehyde solution with 0.1 mM sodium cacodylate buffer (pH 7.4), dehydrated step by step with alcohol using increasing concentration, dried with critical-point drying, and finally sprayed with by metals^12^. The observations were made and photographed with the help of Scanning Electron Microscope (SEM, JSM-6390LV, JEOL Co., Japan) under ×100 and ×400 magnification. The photographs of root hairs obtained were analyzed through the Image J software (http://rsb.info.nih.gov/ij/) for observations on length, diameter and density.

#### Root IAA concentration

Root IAA was extracted as per the protocol of Chen *et al*.^37^ and determined by an Enzyme-Linked Immunosorbent Assay (ELISA), provided by the Engineering Research Center of Plant Growth Regulator, China Agricultural University, Beijing, China.

#### Root IAAO activity

Root IAAO activity was measured using the ELISA assay (BYE97073, Shanghai Bangyi Biotechnology Co. Ltd, China) according to the user’s guide.

#### Root IAA fluxes

A non-invasive micro-test technique (NMT) was used to determine the root IAA flux in the root hair zone (the 3 cm place from the root tip). Non-invasive micro-test system (NMT100 Series, YoungerUSA LLC, Amherst, MA01002, USA; Xuyue (Beijing) Sci. & Tech. Co., Ltd., Beijing, China) with Aset 2.0 (Science Wares, Falmouth, MA, USA) and iFluxes 1.0 software (YongerUSA, LLC, Amherst, MA 01002, USA) were used. Root preparation was made according to the protocol as suggested by Yan *et al*.^38^ and incubated with a balance solution (pH 6.1) containing 0.2 mmol/L KCl and 0.2 mmol/L CaCl_2_ for 10 min. An IAA-sensitive microsensor (Φ2 ± 4 µm, XY-DJ-600, YoungerUSA) was polarized at +700 mV. The IAA sensor was then placed near (2 µm) the surface of the roots. Before testing, the IAA electrode was calibrated with 0, 2, 4, 6, and 8 μM IAA coupled with using linear calibration slope (*R*^2^ > 0.99). A representative plant from each pot was measured once. The IAA flux was calculated using the Fick’s first law of diffusion: *J* = −*D* × Δ*C*/Δ*X*, where *J* is free IAA flux (fmol/cm^2^/s) (positive and negative value were used as efflux and influx, respectively), *D* the molecular diffusion coefficient (7 × 10^−6^ cm^2^/s), Δ*C* the auxin concentration gradient (gmol), and Δ*X* the excursion distance for the microelectrode oscillation (30 μm).

#### Quantitative RT-PCR

Freezed root sample was ground in liquid nitrogen. Root total RNA was extracted using an EASY spin Plus plant RNA kit (RN 38, Aidlab Biotecnolohies Co. Ltd, China). After DNase treatment, total RNA was reversely transcribed to cDNA using the PrimeScript^TM^ RT reagent kit (PK02006, Takara Bio. Inc, Japan). Quantitative real-time PCR (qRT-PCR) were performed using the Power SYBR Green PCR Master Mix kit (Applied Biosystems, CA, USA) on a 7900HT Fast Real-time PCR System (Applied Biosystems, CA, USA). The amplification protocol consists of one cycle of 95 °C for 10 min, followed by 40 amplification cycles of 95 °C for 15 s, 56 °C for 30 s, and 72 °C for 30 s. The primers for selected auxin efflux carriers (*PIN1*, *PIN3*, and *PIN4*), auxin influx carriers (*AUX1*, *LAX1*, *LAX2*, *LAX3*, *ABCB1*, and *ABCB19*), auxin synthesized genes (*TAA1*, *TAR2*, *YUC3*, *YUC4*, *YUC6*, and *YUC8*), and root hair-specific expansin genes (*EXPA4*, *EXPA5*, and *EXPA7*) in the qRT-PCR were shown in Table 2, as per the design from *Citrus sinensis* cDNA sequences (http://citrus.hzau.edu.cn/orange). The relative fold change in gene expression was calculated by the 2^−△△Ct^ method^39^, where the reference gene β-actin was acted as the control.

**Table 2:** Gene-specific primer sequences used in this work for qRT-PCR.

Gene	Accession No.	Forward primer (5′-3′)	Revers primer (5′-3′)
*PtABCB1*	Ciclev10010916m	GAGCCATTCACGCCACTTC	TCTTGTAACCGAGCCTTTGAGC
*PtABCB19*	Ciclev10010931m	GCATGAGTTTGGGTCAGTCTTT	CATCTTCCATTTGTTGGGTCTT
*PtAUX1*	Ciclev10011596m	CTTGACTCTGCCCTATTCATTCTC	TGGACCCAGTAACCCATCAAGC
*PtEXPA4*	Cs7g32410.1	GACCGCCGTACTTCCACTTCTTG	GGAAAGTGCTTGAAACTAAACCCTGAA
*PtEXPA5*	Cs8g18640.1	AACTAACTACACGGAGCTGTGTCTTCT	CGGAGTAATCGCCAGGGAGTCTTG
*PtEXPA7*	Cs5g10000.1	AGGGAACAAGAACAGGATGGATTAGCA	CCAGTTAGCAGGAGCAACATTGTAAGC
*PtLAX1*	Ciclev10031413m	TTGGCGGACATGCAGTGAC	CAGCGGCAGCAGAAGGAAT
*PtLAX2*	Ciclev10028271m	TGTGGGAAGATGGGTAGGGAC	TAGTVATGCTCGCCCACCC
*PtLAX3*	Ciclev10001072m	ATCACTTTCGCTCCTGCTGC	CAAACCCAAATCCCACCACTA
*PtPIN1*	Ciclev10007787m	GCTTTGGCAACAGAAGAGGATT	ATTACACTTGTCGGCGGCATA
*PtPIN3*	Orange1.1g006199m	CATGCCTCCAGCGAGTGTTAT	TGCCACCTGAAAGCGATTAGA
*PtPIN4*	Ciclev10012938m	ATGGGGTTGAAAACGAAGGG	CCTGATAAGTTTCCTCCACACCA
*PtTAA1*	Ciclev10033774m	TTTGAGGCGTTTTGGAGGAA	TTGTTGATTGCTTCAGCGAGTT
*PtTAR2*	Ciclev10020085m	CACACACGGCACACCCCTA	GCCTCCCACTCCCCAGATC
*PtYUC3*	Ciclev10006828m	CCTTCAGGTTTAGCCGTTGC	GGAAGTTTGGAAGTTGGCAGA
*PtYUC4*	Ciclev10008466m	GACCATCTGGGTTAGCCGTTT	GTATTTTGGGAAGTTTTCAGGGA
*PtYUC6*	Ciclev10008473m	GTGGTTGCTAAAGTGGCTGC	GTTGAAGGGGACCCAAAAGA
*PtYUC8*	Ciclev10020503m	GTGATAATGGTAGGGGCAGGA	GAATGGCAGGTGAGGGAGC
*β-actin*	Cs1g05000	CCGACCGTATGAGCAAGGAAA	TTCCTGTGGACAATGGATGGA

### Statistical analysis

The data were statistically analyzed using one-way ANOVA (SAS, version 8.1). Data of root AM colonization were arcsine transformed prior to ANOVA analyses. The Duncan’s multiple range tests were used to compare significant differences among treatments at *P* < 0.05.
